# Differential processing of binocular and monocular gloss cues in human visual cortex

**DOI:** 10.1152/jn.00829.2015

**Published:** 2016-02-24

**Authors:** Hua-Chun Sun, Massimiliano Di Luca, Hiroshi Ban, Alexander Muryy, Roland W. Fleming, Andrew E. Welchman

**Affiliations:** ^1^School of Psychology, University of Birmingham, Birmingham, United Kingdom;; ^2^Center for Information and Neural Networks, National Institute of Information and Communications Technology, and Osaka University, Osaka, Japan;; ^3^Graduate School of Frontier Biosciences, Osaka University, Osaka, Japan;; ^4^School of Psychology, University of Southampton, Southampton, United Kingdom;; ^5^Department of Psychology, Justus-Liebig-Universität Giessen, Germany; and; ^6^Department of Psychology, University of Cambridge, Cambridge, United Kingdom

**Keywords:** surface gloss, material perception, specularity, MVPA, fMRI, binocular cue

## Abstract

The visual impression of an object's surface reflectance (“gloss”) relies on a range of visual cues, both monocular and binocular. Whereas previous imaging work has identified processing within ventral visual areas as important for monocular cues, little is known about cortical areas involved in processing binocular cues. Here, we used human functional MRI (fMRI) to test for brain areas selectively involved in the processing of binocular cues. We manipulated stereoscopic information to create four conditions that differed in their disparity structure and in the impression of surface gloss that they evoked. We performed multivoxel pattern analysis to find areas whose fMRI responses allow classes of stimuli to be distinguished based on their depth structure vs. material appearance. We show that higher dorsal areas play a role in processing binocular gloss information, in addition to known ventral areas involved in material processing, with ventral area lateral occipital responding to both object shape and surface material properties. Moreover, we tested for similarities between the representation of gloss from binocular cues and monocular cues. Specifically, we tested for transfer in the decoding performance of an algorithm trained on glossy vs. matte objects defined by either binocular or by monocular cues. We found transfer effects from monocular to binocular cues in dorsal visual area V3B/kinetic occipital (KO), suggesting a shared representation of the two cues in this area. These results indicate the involvement of mid- to high-level visual circuitry in the estimation of surface material properties, with V3B/KO potentially playing a role in integrating monocular and binocular cues.

surface gloss provides important information about the characteristics of visual objects: for instance, shiny metal objects are usually manufactured recently and have better conductance than rusty metal, whereas fresh apples have glossier skin than rotten ones. However, the estimation of gloss poses a difficult challenge to the visual system: the viewer has to separate the surface properties of the object from information about the illumination and three-dimensional (3D) shape of the object (Anderson 2011). Here, we sought to investigate the neural circuits that play a role in meeting this challenge to estimate gloss.

A number of investigators have studied the neural basis of gloss computations by manipulating the specular and diffuse surface-reflectance properties of objects (Kentridge et al. 2012; Nishio et al. 2012, 2014; Okazawa et al. 2012; Sun et al. 2015; Wada et al. 2014). For instance, functional MRI (fMRI) and single-cell recordings in the macaque brain have demonstrated that gloss information from reflections of the surrounding environment (i.e., specular reflections) is processed along the ventral visual pathway from V1, V2, V3, and V4 to superior temporal sulcus and inferior temporal cortex (Nishio et al. 2012; Okazawa et al. 2012). Similarly, human studies suggested that specular highlight cues to gloss are primarily processed in the ventral processing stream: V4, ventral occipital 1/2 area, lateral occipital (LO) area, collateral sulcus, and posterior fusiform sulcus (pFs) (Sun et al. 2015; Wada et al. 2014). Furthermore, these human studies suggested the involvement of V3B/kinetic occipital (KO) in gloss processing.

This previous work has involved participants looking at (stereoscopically) flat pictorial representations of glossy surfaces. This follows the tradition of psychophysical studies that have identified a number of pictorial signals that could be used to identify surface-reflectance properties (Anderson and Kim 2009; Doerschner et al. 2010, 2011; Fleming et al. 2003; Gegenfurtner et al. 2013; Kim and Anderson 2010; Kim et al. 2011, 2012; Landy 2007; Marlow and Anderson 2013; Marlow et al. 2011; Motoyoshi et al. 2007). For convenience, we will refer to these types of pictorial cues as “monocular,” in the sense that they allow a viewer to gain an impression of surface gloss based on a single view of the stimuli.

In addition to monocular gloss cues, it is clear that potentially important information about surface-reflectance properties comes from binocular cues. In particular, the observation of glossy surfaces binocularly typically results in the two eyes registering a different pattern of reflections, such that specular reflections are displaced away from the physical surface in depth (Blake and Bülthoff 1990; Kerrigan and Adams 2013; Wendt et al. 2008). Past psychophysical work has shown that these binocular signals can strongly modulate the impression of surface gloss (Blake and Bülthoff 1990; Kerrigan and Adams 2013; Muryy et al. 2012; Obein et al. 2004; Sakano and Ando 2010; Wendt et al. 2008, 2010). For instance, Blake and Bülthoff (1990) showed that the simple change in the disparity of a highlight with respect to a physical surface could lead to a considerable change in participants' perceptual impression of surface gloss. Moreover, work characterizing the properties of binocular reflections has shown that the disparities evoked by such stimuli often differ substantially from the disparities evoked when viewing matte objects: disparity gradients are larger, and there can be large, vertical offsets between corresponding image features (Muryy et al. 2013, 2014).

Here, we sought to test for cortical areas engaged by monocular and binocular cues to gloss. The logic of our approach was to contrast stimuli that differed in binocular disparity structure or material appearance and thereby, localize fMRI responses to disparity vs. perceived gloss. An ideal stimulus set would therefore contain the following: *1*) items that had the same material appearance but different disparity structures and *2*) the same disparity but different material appearance, whereas in all cases, keeping other image features identical. Although this ideal scenario is difficult to meet, here, we develop an approach that allows us to implement and address it. In particular, we used a computer graphics rendering approach (Fig. 1) to create stimuli for which we could independently manipulate monocular and binocular gloss cues.

**Fig. 1.**
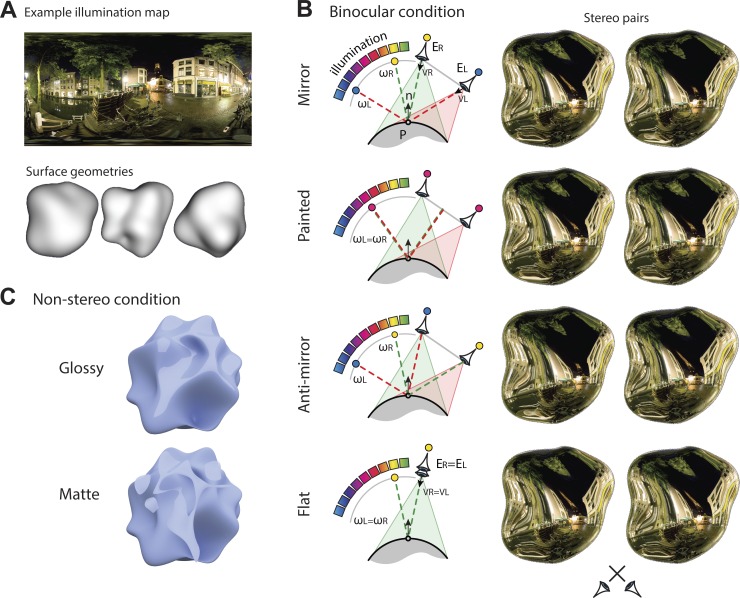
Stimuli used for binocular and nonstereoscopic gloss experiments. *A*: synthetic objects (“potatoes”) were rendered under 3 different illumination maps (Debevec 1998) to create the stimuli. *B*: schematic illustration of the rendering procedure and example stereograms for each condition (cross the eyes to fuse the image pairs). Mirror condition: reflections entering each eye follow the law of specular reflection, creating a physically correct image of a polished object, reflecting its surrounding environment (schematically illustrated using the color spectrum for a single point, P, on the surface of the object). Painted condition: pixel intensities for each location on the surface of the object are determined based on the reflection of a ray cast from midway between the participant's eyes. The object is imaged from the true positions of the 2 eyes, meaning that the environment effectively acts as a texture painted onto the surface of the object. Anti-mirror condition: the reflected ray vectors are reversed for the 2 eyes, so the left eye images a portion of the environment appropriate for the right eye. This alters the disparities produced by reflection, but the object appears glossy. Flat condition: we randomly select the image of 1 eye (the right eye in the example) and present it to both eyes. Objects look flat, and specular reflections have the same apparent depth as the image plane. *C*: an example stimulus in the nonstereoscopic gloss session. Specular components are presented in the Glossy condition, whereas in the Matte condition, the specular components are rotated by 45° in the image plane, making the object appear matte.

We manipulated the rendering process to change the locations from which pixel intensities are determined, while keeping the viewing position constant [see Muryy et al. (2014) for a detailed description]. This allowed us to create four binocular conditions. First, we used physically correct rendering of objects with mirrored surfaces, reflecting a natural scene (Mirror, Fig. 1*B*). Second, we created a “painted” condition, in which the reflections were “stuck” onto the surface of the object. This had the effect that monocular features were almost identical to a glossy object, but when stimuli were viewed stereoscopically, the object appeared matte [Muryy et al. (2013); see also Doerschner et al. (2011) for the analogous case with motion]. Third, we modified the rendering process to create physically incorrect specular reflections (Anti-mirror, Fig. 1*B*). These stimuli had different overall disparity values but nevertheless, evoked an impression of surface gloss. Finally, we presented the same image to the two eyes, creating the impression of a stereoscopically flat object, for which gloss was defined solely by monocular cues (Fig. 1*B*). We thereby sought to test for neural responses relating to changes in binocular signals vs. the perceptual interpretation of surface material properties. In addition, to draw comparisons with neuronal responses to gloss defined by monocular cues, we measured fMRI responses when participants viewed stimuli, for which we used an image-editing technique to alter the impression of surface gloss (Fig. 1*C*). In this way, we aimed to reveal common responses to gloss defined by differences in monocular and binocular cues.

## METHODS

### Participants

Twelve participants with normal or corrected-to-normal vision took part in the experiment. One was an author (H.-C. Sun), and the remainder were naïve. Three were men, and age ranged between 19 and 39 yr. Participants were screened for normal stereoacuity and MRI safety. They provided written, informed consent. All participants took part in three fMRI sessions: one binocular gloss session, one nonstereoscopic gloss session (see *Stimuli* and *Design and Procedure*), and one localizer session (see *ROI definition*). The study was approved by the Science, Technology, Engineering and Mathematics Ethical Review Committee of the University of Birmingham. Nonauthor participants received course credits or monetary compensation.

### Apparatus and Stimuli

#### Apparatus.

Stimulus presentation was controlled using Matlab (MathWorks, Natick, MA) and Psychtoolbox (Brainard 1997; Pelli 1997). The stimuli were back projected by a pair of projectors (JVC D-ILA-SX21) onto a translucent screen inside the bore of the magnet. To present stereoscopic stimuli, the projectors were fitted with spectral comb filters (Infitec, Gerstetten, Germany) [see Preston et al. (2009)]. This presentation technique allows stereoscopic presentation of color images, with only slight differences in the color spectra presented to each eye, and low crosstalk between the two eyes' views. Participants viewed the stimuli binocularly via a front-surface mirror fixed on the head coil with a viewing distance of 65 cm. In the nonstereoscopic gloss session, participants viewed stimuli (binocularly) without wearing the Infitec glasses. Luminance outputs from the projectors were measured using Admesy Brontes-LL colorimeter (Ittervoort, Netherlands) and then linearized and equated for the red-green-blue channels separately with Mcalibrator2 (Ban and Yamamoto 2013). Participant responses during the scan were collected using an optic fiber button box.

#### Stimuli.

A central fixation square (0.5° side length) was displayed in the background to provide a constant reference to promote correct eye vergence. We performed the experiment in two sessions: a binocular gloss session and a nonstereoscopic gloss session. For the binocular gloss session, we used Matlab to create three different 3D objects [created by randomly distorted spheres, that look like potatoes at arms' length (Muryy et al. 2013, 2014)]. The rendering procedure involved using objects with known surface geometries presented at a viewing distance of 65 cm (Fig. 1*A*). The objects had perfectly specular surfaces and reflected one of three different spherical illumination maps [extracted from Debevec (1998)], which for rendering purposes, were located at optical infinity (Fig. 1*A*). The rendered images produced objects that were ∼7° in diameter. These were presented at the center of the screen, with ±0.4° jitter from the center to reduce the buildup of adaptation across repeated presentations at the center of the screen.

To produce stimuli for the four experimental conditions (mirror, painted, anti-mirror, flat) in the binocular gloss session, we made subtle modifications to the stimulus-rendering process [for full details and mathematical implementation, see Muryy et al. (2014)]. In particular, under standard mirror reflection (Fig. 1*B*), stimuli are rendered by finding the pixel value of point *P* in the image of left eye (*E*_*L*_) and right eye (*E*_*R*_) by reflecting the viewing vectors from left eye (*V*_*L*_) and right eye (*V*_*R*_) around the surface normal (*n*) to calculate the reflected ray vectors *ω*_*L*_ and *ω*_*R*_ [e.g., *ω*_*L*_ = 2 (*n V*_*L*_) *n* + *V*_*L*_]. These point to particular image intensities in the spherical illumination map, determining the pixel intensities that should be presented to *E*_*L*_ and *E*_*R*_ (see Fig. 1*B* for an illustration of this process). With the use of computer graphics, we changed the locations from which the objects are imaged for the purpose of defining the pixel intensities of the object, while keeping the stereoview frustum constant (Fig. 1*B*) [see Muryy et al. (2014)]. This allowed us to manipulate the stereoscopic information from the reflections to create four different conditions, while leaving monocular images almost constant.

**Fig. 2.**
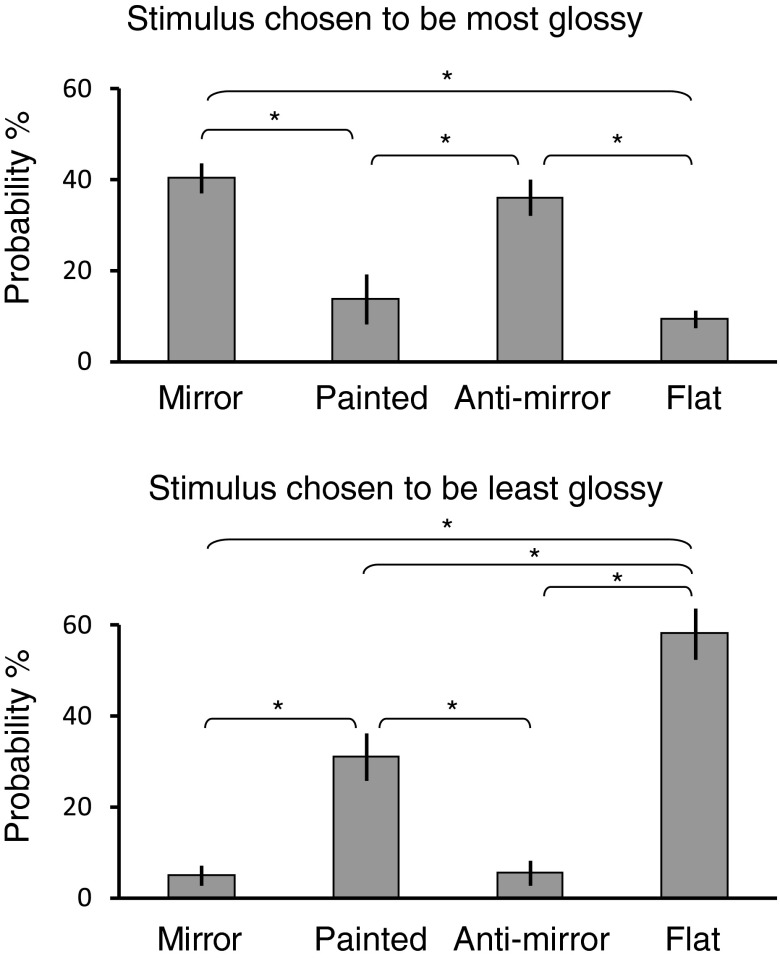
Results of psychophysical ratings of perceived gloss for the different binocular conditions. Participants (*n* = 6; different from the participants of scan sessions) were presented with 4 pairs of stereo stimuli (corresponding to the 4 conditions) concurrently on a screen viewed with 3D prism glasses (NVP3D) in the laboratory. The shape and illumination of each stimulus pair were randomly chosen from the 3 different potato shapes and the 3 different illumination maps described in Fig. 1. Participants were asked to choose the most and the least glossy object by pressing numerical keys that correspond to the position of the 4 stereo stimuli on the screen. Judgments were blocked into 180 trials, with block order counterbalanced across participants. The probability of choosing each condition was averaged across participants. Bar graphs show mean selection probability ±1 SE. A 1-way repeated-measures ANOVA (mirror, painted, anti-mirror, flat) was significant for both blocks (*F*_3,15_ = 12.0, *P* < 0.001 for most glossy block; *F*_3,15_ = 27.3, *P* < 0.001 for least glossy block). *P < 0.05, significant differences based on Tukey's honest significant difference (HSD) post hoc tests.

Specifically, first, in the mirror condition (Fig. 1*B*), stimuli are generated following the normal specular reflection, creating the impression of a mirrored object. Second, in the painted condition (Fig. 1*B*), the specular reflections act like a texture and are effectively stuck onto the surface of the object. This means that the specular reflections have the same stereoscopic depth as the object's surface, although the images still contain classic monocular signals to reflection, such as the distortions of the surrounding illumination map. In the painted case, the stereoscopic information largely overrides these monocular cues, greatly reducing the perception of surface gloss (Fig. 2). Third, in the anti-mirror condition (Fig. 1*B*), we reversed the locations from which image intensities in the environment are determined between the two eyes. This leads to a considerable change in the disparity structure of the images (Muryy et al. 2013); nevertheless, the stimuli are perceived to have a similar glossy appearance to that of a correctly rendered mirror (Muryy et al. 2012) (Fig. 2). Finally, we created a flat condition (Fig. 1*B*), in which the same image of the object was presented to both eyes, again reducing participants' overall impression of gloss (Fig. 2).

To ensure generality in identifying signals related to surface appearance, we used a different set of stimuli in the nonstereoscopic gloss session. In particular, we used single-view renderings of 3D objects (3 different shapes) generated in Blender 2.67a (The Blender project: http://www.blender.org/; Stichting Blender Foundation, Amsterdam, The Netherlands). Participants were presented stimuli in four conditions [Glossy, Matte, Rough, and Textured; see Sun et al. (2016)]. Only data from the Glossy and Matte conditions are presented here. The Rough and Textured conditions are not directly relevant to the current study. To generate the Glossy and Matte stimuli, we first rendered the objects with a specular surface component. We then edited the images in Adobe Photoshop, using the “color range” tool to extract the portions of the objects corresponding to specular reflections (i.e., lighter portions of the shape in Fig. 1*C*, where fuzziness parameter of the color range tool was set to 40 to isolate the specular highlights). We then pasted these highlights onto a rendering of the object produced with no specular surface reflection. When pasted into the “correct” locations (i.e., those that contained highlights for the specular surface), the object appeared glossy (Fig. 1*C*); however, when rotated 45° in the image plane, the surface no longer appeared glossy (Fig. 1*C*; Wilcoxon signed-rank test, two-tailed, *n* = 7, W = 26, *P* < 0.05). This difference in appearance between the two conditions is likely to be due to the incoherence between the position/orientation of the highlights and the contextual information about shape and illumination (Anderson and Kim 2009; Kim et al. 2011; Marlow et al. 2011).

Note that the basic appearance of the stimuli is (deliberately) quite different for the binocular (Fig. 1*B*) and nonstereoscopic (Fig. 1*C*) imaging sessions, as we wished to test for generalization of the impression of gloss that could not be ascribed to simple image features (e.g., contours) or the overall 3D shape. Moreover, note that we did not directly compare brain activity between the two types of stimuli; rather, we looked for generalization across contrasts conducted within each stimulus set (i.e., “gloss vs. matte” generalized to “mirrored vs. painted”).

#### MRI data acquisition.

A 3 Tesla Philips Achieva scanner with an eight-channel phase-array head coil was used to obtain all MRI images at the Birmingham University Imaging Centre. Functional whole-brain scans with an echo-planar imaging (EPI) sequence [axial 32 slices, repetition time (TR) 2,000 ms, echo time (TE) 35 ms, voxel size 2.5 × 2.5 (inplane) × 3 (thickness) mm, flip angle 80°, matrix size 96 × 94] were obtained for each participant. The EPI images were acquired in an ascending, interleaved order for all participants. The same sequence was used in both sessions. T1-weighted, high-resolution anatomical scans (sagittal 175 slices, TR 8.4 ms, TE 3.8 ms, flip angle 8°, voxel size 1 mm^3^) were also obtained to reconstruct cortical surfaces of individual participants and to achieve precise coregistrations of EPI images onto individual anatomical spaces.

### Design and Procedure

**Fig. 3.**
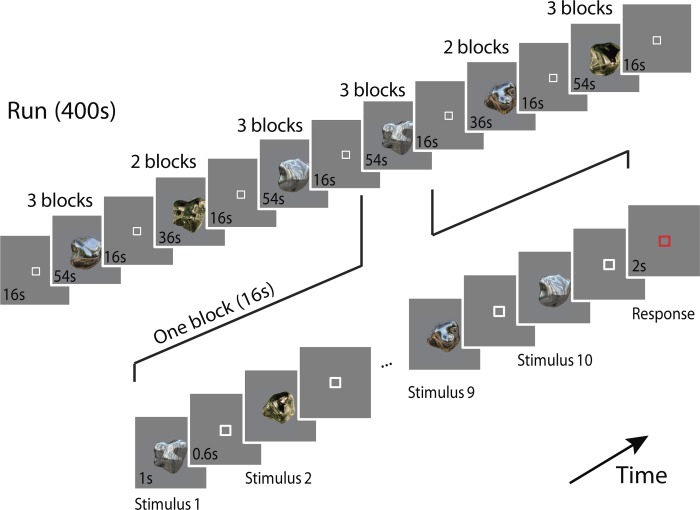
The stimulus presentation protocol in binocular gloss session for 1 scan. On each run, 23 blocks were presented (16 s + 2 s response time each), including 7 fixation blocks and 16 experimental blocks. During each experimental block, stimuli were presented for 1,000 ms with a 600-ms interstimulus interval (ISI). Participants were instructed to detect stimuli that differed from the others in terms of glossiness (oddball detection task for glossiness).

A block design was used in both sessions. Each session took ∼1.5 h, during which each participant completed in 7–10 runs for the binocular gloss session and 8–10 runs for the nonstereoscopic gloss session (depending on setup time and the participants' needs to rest between scans). The run length was 400 and 368 s for the binocular and nonstereoscopic gloss sessions, respectively. Each run started with four dummy scans to prevent startup magnetization transients and consisted of 16 experimental blocks, each lasting 16 s. There were four block types (i.e., 1 for each condition), repeated four times in a run. In each block of the binocular gloss session, 10 objects were presented in a pseudo-random order. Stimuli were presented for 1,000 ms with a 600-ms interstimulus interval (ISI). Participants were instructed to maintain fixation and perform an oddball task for glossiness judgments. Specifically, at the end of each block (signaled to the participants by a change in the fixation marker), participants had to indicate if all of the presented objects had the same glossiness (i.e., all matte or all glossy) or whether one of the presented objects differed in gloss. They had 2 s to make their response before the next block began. They were able to perform this task well [mean discriminability (d′) = 2.04; SE = 0.31]. Five, 16 s fixation blocks were interposed after the 3rd, 5th, 8th, 11th, and 13th stimulus blocks to measure fMRI signal baseline. In addition, 16 s fixation blocks were interposed at the beginning and at the end of the scan, making a total of seven fixation blocks during one experimental run. An illustration of the scan procedure is provided in Fig. 3. In the nonstereoscopic gloss session, stimuli were presented for 500 ms with a 500-ms ISI. Participants were instructed to maintain fixation and perform a one-back matching task, whereby they pressed a button if the same image was presented twice in a row. They were able to perform this task well (mean d′ = 2.03; SE = 0.10). Other details were the same as for the binocular gloss session.

### Data Analysis

#### fMRI data processing.

The basic data processing procedures for both the binocular and the nonstereoscopic gloss sessions are identical to our previous studies (Sun et al. 2015, 2016). To summarize the procedure, we computed the global signal variance of the blood oxygenation level-dependent signal for each run using the whole-brain average of activity across volumes. If this exceeded 0.23%, then the scan run was excluded from further analysis to avoid the influence of scanner drifts, physiological noise, or other artifacts (Junghöfer et al. 2005). On this basis, 17/146 runs and 6/118 runs across 12 participants for binocular and nonstereoscopic gloss sessions, respectively, were excluded from further analysis.

#### ROI definition.

A total of 15 regions of interest (ROIs) was defined. For all participants, V1, V2, V3v, V4, V3d, V3A, V3B/KO region, human motion complex (hMT+)/V5, LO region, and pFs were defined by localizers in a separate session, as in previous studies (Ban et al. 2012; Dövencioğlu et al. 2013; Murphy et al. 2013; Sun et al. 2015). For 7 of the 12 participants, higher dorsal areas V7, ventral intraparietal sulcus (VIPS), parieto-occipital IPS (POIPS), dorsal IPS medial (DIPSM), and dorsal IPS anterior (DIPSA) were also defined by a localizer, in which a random-dot stereogram with 3D structure from motion information was contrasted with moving dots without stereogram and structure from motion information (Orban et al. 2006, 1999). For the other five participants, V7 was identified as anterior and dorsal to V3A and other dorsal areas, defined according to Talairach coordinates (x,y,z = [30, −78, 27] for right VIPS; [−27, −72, 30] for left VIPS; [24, −75, 45] for right POIPS; [−18, −72, 54] for left POIPS; [18, −60, 63] for right DIPSM; [−15, −63, 60] for left DIPSM; [39, −36, 54] for right DIPSA; [−36, −48, 60] for left DIPSA), and draws around general linear model *t*-value maps that had a *t* value greater than zero for the contrast of “all experiment conditions vs. fixation block” (Dövencioğlu et al. 2013; Murphy et al. 2013; Orban et al. 2003).

#### Additional fMRI analysis.

We used multivoxel pattern analysis (MVPA) to compute prediction accuracies for the experimental conditions. We selected voxels by first computing the contrast “all experimental conditions vs. fixation” and then selecting the top 250 voxels from this contrast within each ROI of each individual participant (Ban et al. 2012). If a participant had <250 voxels in a particular ROI, then we used the maximum number of voxels that had *t* > 0. After selecting the voxels, we extracted the time series (shifted by 4 s to account for the hemodynamics response delay) and converted the data z-scores. Then, the voxel-by-voxel signal magnitudes for a stimulus condition were obtained by averaging over eight time points (TRs; = 1 block) separately for each scanning run. To remove baseline differences in the response patterns between stimulus conditions and scanning runs, we normalized by subtracting the mean for each time point. To perform the MVPA, we used a linear support vector machine (SVM), implemented in the LIBSVM toolbox (http://www.csie.ntu.edu.tw/∼cjlin/libsvm) (Chang and Lin 2011) to discriminate the different conditions in each ROI. In the training phase, 24 response patterns for each stimulus condition were used as a training dataset for those participants that completed 7 runs, and 36 response patterns were used for those who completed 10 runs. Then, four response patterns for each condition were classified by the trained classifier in the test phase. These training/test sessions were repeated and validated by a leave-one-run-out cross-validation procedure. The ROI-based prediction accuracy for each participant was defined as a mean of these cross-validation classifications. In situations where there were different numbers of samples between two conditions in a contrast (e.g., mirror and anti-mirror vs. painted), we used balanced weight vectors for each class by adjusting the *j* parameter in the LIBSVM toolbox to eliminate bias from a different number of samples in the training dataset. We also used a searchlight classification analysis approach (Kriegeskorte et al. 2006), whereby we defined a spherical ROI with 8 mm radius and moved it through the entire volume of cortex with masking volumes so that the searchlight sphere only captured gray-matter voxels. For each location, we recomputed the SVM classification analysis.

## RESULTS

**Fig. 4.**
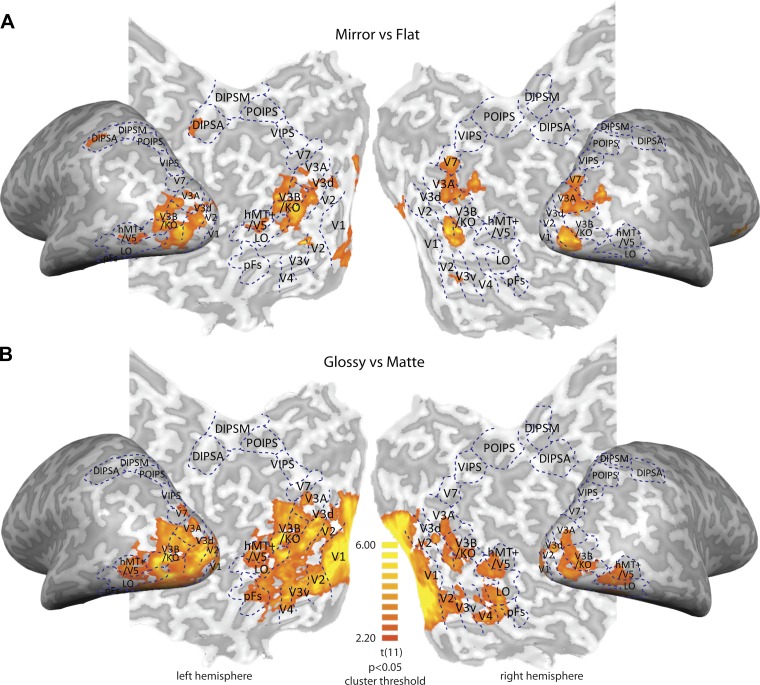
Searchlight classification analysis results for binocular (*A*) and nonstereoscopic (*B*) gloss conditions across 12 participants. The color code represents significant *t* value of Mirror vs. Flat and Glossy vs. Matte classification accuracies in *A* and *B*, respectively (testing against chance level 0.5). Blue, dashed lines are the ROI boundaries that we defined with independent localizer scans. The significance level is *P* < 0.05, with cluster-size thresholding 25 mm^2^. Regions with significant results are presented on the flat maps of 1 representative participant. Note that since classification results are averaged across participants and then presented on the flat maps of 1 representative participant, individual ROI boundaries may not perfectly fit the group level.

**Fig. 5.**
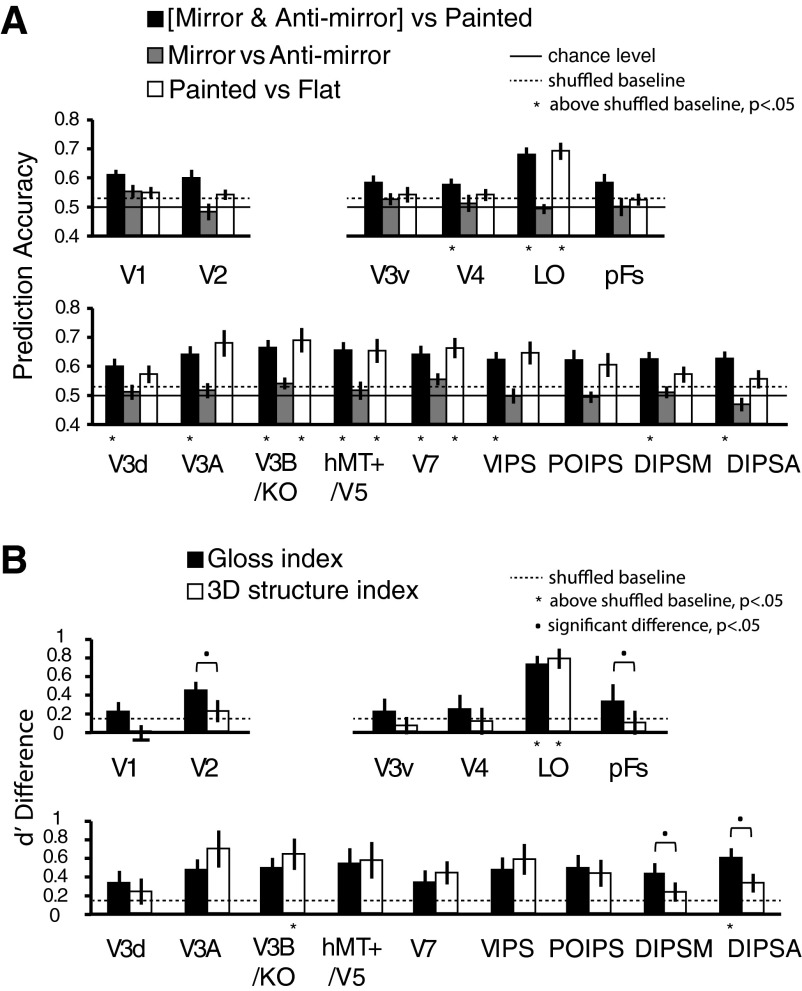
MVPA prediction performance across 12 participants for [mirror and anti-mirror] vs. painted (black bars), mirror vs. anti-mirror (gray bars), and painted vs. flat (white bars; *A*). The bars reflect mean prediction accuracy with ±1 SE. Solid horizontal lines represent chance performance for the binary classification (0.5); dotted horizontal lines represent the upper 95th percentile with permutation tests (1,000 repetitions for each ROI of each participant with randomly shuffling stimulus condition labels per test). The one-tailed, 95% boundaries of accuracy distributions were averaged across all ROIs, which were 52.52% for [mirror and anti-mirror] vs. painted, 53.11% for mirror vs. anti-mirror, and 53.13% for painted vs. flat. Asterisks at the bottom of the bars represent accuracies significantly above the shuffled baseline (*P* < 0.05, one-tailed, Bonferroni corrected). *B*: d′ difference between [mirror and anti-mirror] vs. painted and mirror vs. anti-mirror classification is used as a Gloss index. The d′ difference between painted vs. flat and mirror vs. anti-mirror is used as a 3D structure index. Dotted horizontal lines represent the upper 95th percentile of permutation tests (1,000 repetitions). Asterisks at the bottom of the bars indicate that the index was significantly above the shuffled baseline (*P* < 0.05, one-tailed, Bonferroni corrected). Black dots above bar pairs represent significant difference between the 2 indexes (Tukey's HSD post hoc test at *P* < 0.05).

To test for visual responses related to binocular and monocular cues to gloss, we first identified ROIs within the visual and parietal cortex using independent localizer scans (Fig. 4). We then used MVPA to test for responses related to the impression of glossy vs. matte surfaces. In particular, we used responses in different experimental conditions to understand how fMRI signals might relate to changes in the material appearance of the viewed object vs. changes in the disparity-defined depth structure. To this end, we concentrated on three main contrasts (Fig. 5*A*). First, we tested for responses related to surface gloss, contrasting the mirror and anti-mirror conditions [both perceived as glossy (Fig. 2), and their averaged, overall disparity is (approximately) the same as in the painted condition] against the painted object (perceptually matte). Second, we performed a contrast between the mirror and anti-mirror conditions; the logic of this contrast is that although both appear glossy, the raw disparity composition of the shapes is quite different. Third, we contrasted the painted and flat conditions, which provides the maximal change in 3D shape, whereas both are interpreted as not evoking a strong impression of gloss (Fig. 2). In the extreme scenario of a cortical region specialized for processing surface material, we would expect to be able to decode glossy vs. matte renderings of the stimuli but not the difference between mirror and anti-mirror conditions or the difference between the painted and flat conditions.

We found that we were able to predict the stimulus from the fMRI data at levels reliably above chance (*P* < 0.05, one-tailed, Bonferroni corrected) in multiple ROIs (V4, LO, V3d, V3A, V3B/KO, hMT+/V5, V7, VIPS, DIPSM, DIPSA) when contrasting the mirror and anti-mirror conditions against their painted counterparts (Fig. 5*A*). This suggests widespread sensitivity to differences in the material appearance, whether or not the specular reflections are physically correct. Considering the differences between the mirror and anti-mirror conditions (Fig. 5*A*), we were not able to predict the stimuli reliably in any ROI. This failure to decode differences between the two conditions might suggest widespread responses that respond to glossy appearance and thus do not differentiate between the mirror and anti-mirror conditions. Nevertheless, the interpretation of such a null result requires caution: disparity differences between the stimuli may have been insufficient to support decoding, or the size of the differences between mirror and anti-mirror conditions may have been dwarfed by the disparity differences between the different 3D shapes that were presented. Finally, the contrast in the painted and flat conditions (Fig. 5*A*) revealed above chance-prediction accuracies in V3B/KO, hMT+/V5, V7, and LO (*P* < 0.05, one-tailed, Bonferroni corrected). The decoding performance in this condition allows us to identify areas sensitive to changes in the 3D structure of the shapes. The result is consistent with previous work, suggesting sensitivity to disparity-defined depth in these areas (Ban et al. 2012; Dövencioğlu et al. 2013; Murphy et al. 2013).

To facilitate comparison of performance between conditions, we calculated a “3D structure index” to examine decoding performance that could be attributed to information about 3D shape. We expressed prediction performance in units of d′ and contrasted performance for the mirror vs. anti-mirror condition with the painted vs. flat condition, based on a simple subtraction. The logic of this contrast is that for both sets of comparisons, there is minimal difference in the material appearance of the shapes, so the contrast reflects differences in the 3D structure of the shapes in both conditions. We also created a “Gloss index” by contrasting performance in the mirror vs. anti-mirror contrast with the [mirror and anti-mirror] vs. painted classification. The logic of this contrast is to compare similarly glossy objects (with different disparity information) with differentially glossy objects (with different disparity information). The formulas of the two indices are presented as the following: 3D structure index = d′(painted vs. flat) − d′(mirror vs. anti-mirror); Gloss index = d′(mirror and anti-mirror vs. painted) − d′(mirror vs. anti-mirror).

We used mirror vs. anti-mirror as a baseline for normalizing 3D structure index and Gloss index, because in this contrast, both conditions have the same visual appearance (glossy) and similar 3D structure. The comparison between the two indices is suggestive of whether a brain area is more specialized for gloss processing or 3D structure processing. We present the two indices across all ROIs in Fig. 5*B*. We first considered whether the indices are significantly above chance level (*P* < 0.05, one-tailed, Bonferroni corrected), using permutation tests to calculate 95% shuffled baseline of d′ difference for Gloss index (0.14) and 3D structure index (0.16). We found that the Gloss index was significantly above chance in DIPSA (*t*_11_ = 4.4, *P* < 0.01) and LO (*t*_11_ = 5.3, *P* < 0.01), suggesting that signals in these areas are discriminable based on gloss information. For the 3D structure index, we found sensitivity significantly above chance in V3B/KO (*t*_11_ = 3.5, *P* < 0.05) and LO (*t*_11_ = 4.1, *P* < 0.05). These results suggest that LO processes information relevant to both 3D structure and material properties.

**Fig. 6.**
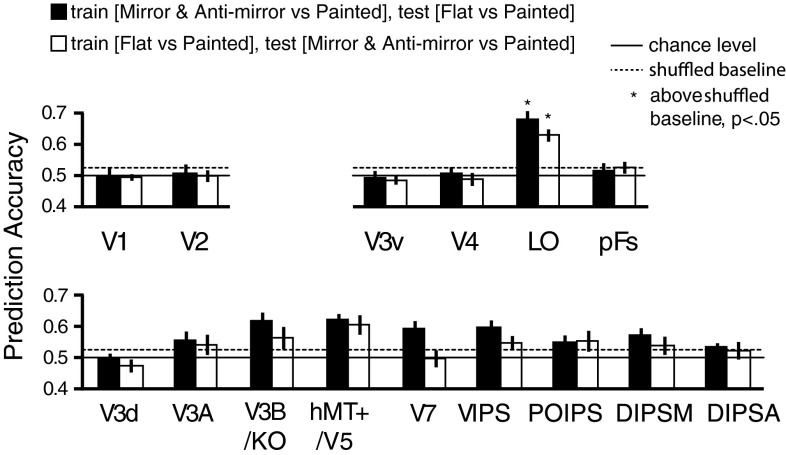
MVPA prediction performance across 12 participants for transfer analysis between [mirror and anti-mirror vs. painted] and [flat vs. painted]. We trained the SVM classifier to discriminate [mirror and anti-mirror vs. painted] and tested whether it is distinguishable for [flat vs. painted] (black bars). We also tested the transfer effect in the other way (white bars). The bars reflect mean classification accuracy with ±1 SE. Solid horizontal lines represent chance performance 0.5 for the binary classification. Dotted horizontal lines represent the upper 95th percentile with permutation tests (1,000 repetitions). The one-tailed, 95% boundaries of accuracy distributions for black bars were 52.24% and 53.17% for white bars. Asterisks at the top of the bars represent that the accuracies were significantly above the shuffled baseline (*P* < 0.05, one-tailed, Bonferroni corrected).

We next sought to compare the indices against each other. To this end, we ran a 2 (Gloss index and 3D structure index) × 15 (ROIs) repeated-measures ANOVA. This indicated a main effect of ROI (*F*_14,154_ = 2.5, *P* < 0.01) and importantly, a significant interaction with index (*F*_14,154_ = 2.8, *P* < 0.01). We then used post hoc contrasts to test the differences between the indices in each ROI. We found a significantly higher Gloss index in V2, pFs, DIPSM, and DIPSA, suggesting areas preferentially engaged in the processing of material properties (Fig. 5*B*). It is reassuring to note that areas V2 and pFs were previously found to be involved in the processing of information about specular reflectance from monocular cues (Sun et al. 2015; Wada et al. 2014), suggesting that they represent general information about surface gloss regardless of the source. In summary, LO appears to process both surface properties and 3D structure information, whereas V2, pFs, DIPSM, and DIPSA selectively process surface properties. Transfer analysis between [mirror and anti-mirror vs. painted] and [flat vs. painted] suggested that the processing of surface properties and 3D structure information involves the same voxels in LO (see Fig. 6).

To ensure that we had not missed any important loci of activity related to gloss or structure, we used a searchlight classification analysis (Fig. 4*A*). This confirmed that locations identified by the searchlight procedure fell within those we had sampled using our ROI localizer approach.

**Fig. 7.**
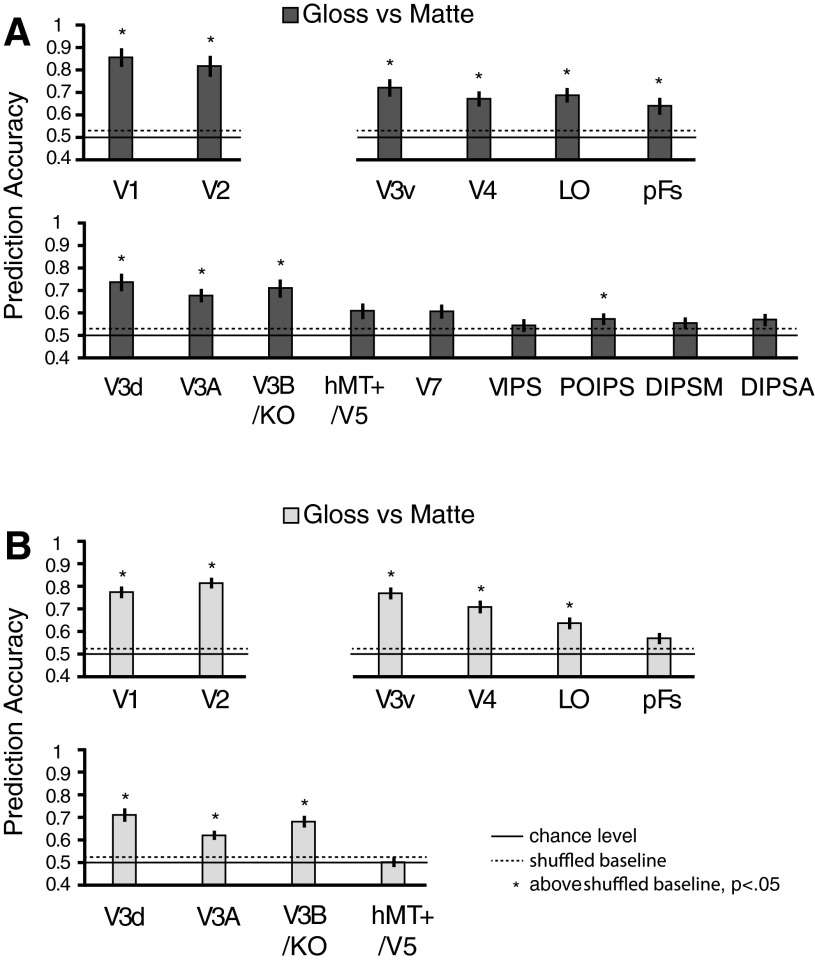
MVPA prediction performance for Glossy vs. Matte in nonstereoscopic gloss session in the current study (*A*) and in our previous study (Sun et al. 2015) with a group of 15 participants (*B*). The bars reflect mean classification accuracy with ±1 SE. Solid horizontal lines represent chance performance 0.5 for the binary classification. Dotted horizontal lines represent the upper 95th percentile with permutation tests (1,000 repetitions). The one-tailed, 95% boundaries of accuracy distributions in *A* were 52.79% and 52.39% in *B*. Asterisks at the top of the bars represent that the accuracies were significantly above the shuffled baseline (*P* < 0.05, one-tailed, Bonferroni corrected). Higher dorsal areas (V7–DIPSA) were not defined in *B*, as the parietal localizer was not applied in that study.

In addition to making measurements of binocularly defined gloss, we used an image-editing procedure to alter the impression of gloss evoked by monocular cues (Fig. 1*C*). As an initial analysis of the fMRI responses evoked by viewing these stimuli, we tested for the ability of an MVPA classifier to discriminate glossy vs. matte stimuli. Figure 7*A* shows the classification results of Glossy vs. Matte stimuli. We found widespread performance above chance (*P* < 0.05, one-tailed, Bonferroni corrected) when comparing between glossy and matte versions of the stimuli (V1, V2, V3v, V4, LO, pFs, V3d, V3A, V3B/KO, POIPS). This was consistent with an MVPA of data collected in a previous study (Sun et al. 2015) that contrasted objects rendered with different surface-reflection parameters to alter perceived gloss (Fig. 7*B*). This also indicates that the additional conditions (Rough and Textured) that were used in the nonstereoscopic gloss session had a very limited effect on gloss processing, because the results are consistent with our previous study (Sun et al. 2015), which did not contain Rough and Textured conditions.

Considering the nonstereoscopic gloss results together with the preceding binocular gloss results suggests that some cortical areas (i.e., V3d, V3A, V3B/KO, V4, LO) support the decoding of both monocular and binocular gloss cues. However, our critical interest was whether the same neural populations (as sampled by voxels) were involved in processing of both binocular and monocular gloss cues. To examine this issue, we performed a transfer analysis to test whether training a classifier on gloss defined by monocular cues (nonstereoscopic imaging session) would support predictions for fMRI responses evoked by binocular cues (and vice versa). Our expectation was that a cortical area that shows transfer in both directions would suggest an area intricately involved in processing gloss, regardless of its image source.

**Fig. 8.**
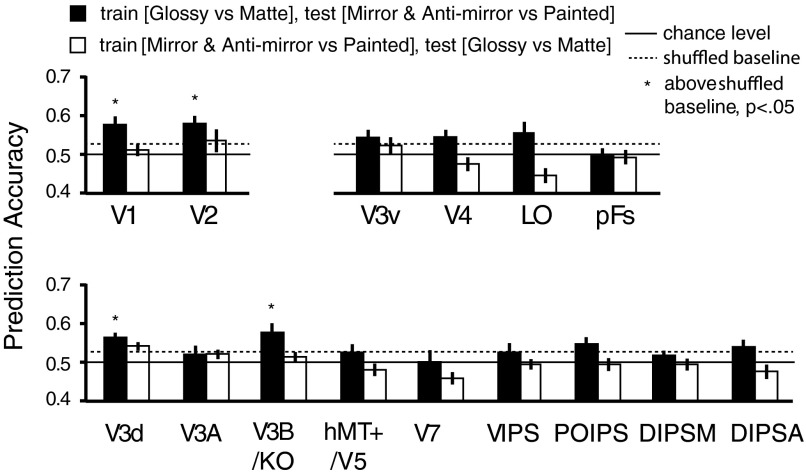
MVPA prediction performance across 12 participants for the transfer analysis between binocular and monocular gloss cues. We trained the SVM classifier to discriminate [Glossy *vs*. Matte] conditions in nonstereoscopic gloss session and tested whether it could predict [mirror and anti-mirror vs. painted] in the binocular gloss session (black bars). We also tested the transfer effect the other way (white bars). The bars reflect mean classification accuracy with ±1 SE. Solid horizontal lines represent chance performance 0.5 for the binary classification. Dotted horizontal lines represent the upper 95th percentile with permutation tests (1,000 repetitions for each ROI). Asterisks at the top of the bars represent that the accuracies were significantly above shuffled baseline (*P* < 0.05, one-tailed, without correction).

**Fig. 9.**
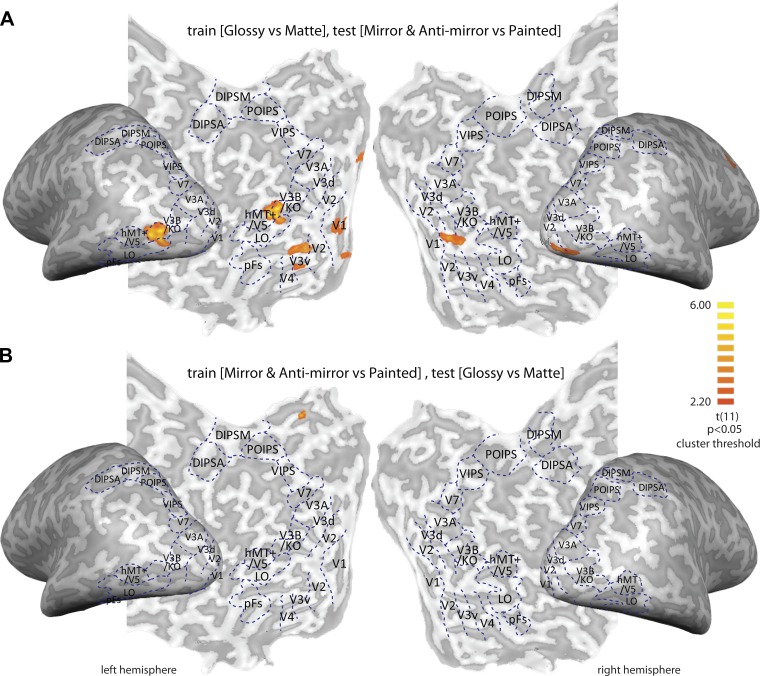
Searchlight transfer analysis results. *A*: we trained the SVM classifier to discriminate [Glossy vs. Matte] conditions in the nonstereoscopic gloss session and then tested [mirror and anti-mirror vs. painted] in the binocular gloss session. *B*: we tested for transfer in the opposite direction. The color code represents the *t* value against chance level (0.5), with 25 mm^2^ cluster-size thresholding. Significant transfer is found, primarily by training on nonstereoscopic gloss cues and subsequently testing on binocular information but not in the opposite direction.

We first trained the SVM classifier to discriminate [Glossy vs. Matte] conditions in the nonstereoscopic gloss session and then tested whether the classifier could discriminate [mirror and anti-mirror vs. painted] activation in the binocular gloss session. We found significant transfer from monocular to binocular gloss in areas V1, V2, V3d, and V3B/KO (Fig. 8). We then tested whether there was transfer from binocular gloss to monocular gloss but found no evidence for transfer in this direction (Fig. 8). As a follow-up analysis, we also conducted a searchlight classification analysis, in case our ROI approach did not capture important loci of activity. This analysis confirmed our choice of ROIs and reconfirmed that whereas we observed transfer from monocular to binocular gloss cues (Fig. 9*A*), we did not observe transfer from binocular to monocular gloss cues (Fig. 9*B*).

## DISCUSSION

Here, we sought to test for cortical areas involved in the processing of gloss from binocular and monocular cues to surface material. We sampled fMRI activity from across the visual processing hierarchy and contrasted fMRI responses in conditions that evoked different impressions of surface gloss. We found that ventral area LO supported the decoding of information about both the material properties of objects and 3D structure. By contrast, we found that differences in gloss were more discriminable than differences in disparity-defined shape based on fMRI responses in DIPSA. We contrasted responses to monocular and binocular signals to gloss, finding differential involvement of areas within the dorsal and ventral streams. Importantly, V3B/KO appeared to be involved in the processing of both types of information. This was supported by a transfer analysis that showed that binocularly specified gloss could be decoded using an algorithm trained on differences in perceived gloss specified by monocular features. These results point to the involvement of both ventral and dorsal brain areas in processing information related to gloss, with an intriguing confluence in area V3B/KO that has previously been associated with the processing of the 3D structure.

Our approach to investigating binocular cues to gloss was to make subtle modifications to the rendering process so that low-level image statistics were almost identical between different conditions. This allowed us to test for the neural processing of binocular signals to surface-reflectance properties, which are likely to interact with the processing of monocular cues to gloss (such as the luminance intensity of specular reflections and their contrast and spatial frequency) (Marlow and Anderson 2013; Marlow et al. 2012; Motoyoshi et al. 2007; Sharan et al. 2008). To test the impression of gloss from monocular cues, we also used a simple image-editing technique that altered participants' impressions of gloss by rotating specular highlight components in the image plane. This broke the relationship between surface curvatures specified by the image and the location of reflections (Fig. 1*C*) and ensured that low-level image features were near identical (Anderson and Kim 2009; Kim et al. 2011; Marlow et al. 2011). This is a different procedure to that used in previous studies that used spatial scrambling, phase scrambling, or changing overall luminance (Okazawa et al. 2012; Sun et al. 2015; Wada et al. 2014). It is reassuring that the results of this manipulation (Fig. 7*A*) converge with a comparable analysis of results from a previous study that used image scrambling (Fig. 7*B*) (Sun et al. 2015). In particular, both datasets indicate that monocular gloss cues are processed in ventral areas, as well as in dorsal areas V3d, V3A, and V3B/KO.

More broadly, our results suggest that gloss-related signals are processed in earlier visual areas (V1, V2, V3d, V3v) and ventral visual areas (V4, LO, pFs), consistent with previous findings (Okazawa et al. 2012; Wada et al. 2014). We provide converging evidence in line with two previous studies (using a different approach to generate stimuli) that human V3B/KO is involved in gloss processing (Sun et al. 2015; Wada et al. 2014). In addition, our results indicate that higher dorsal area POIPS supports the decoding of monocular gloss cues (Fig. 7*A*). This is not something that has been found before (Sun et al. 2015; Wada et al. 2014). It is possible that our use of MVPA to analyze these data provides a more sensitive tool to reveal representations that were not detected using the standard general linear model contrasts in previous work. However, it is also possible that our image-editing technique evoked the impression of surface occlusion that increased the complexity of the viewed shape and may have promoted subtle differences in the degree to which the stimuli engaged the participants' attention.

It is informative to compare the results we obtained in the nonstereoscopic and binocular gloss imaging sessions. Results from the nonstereoscopic gloss manipulations indicated responses in V1 and V2 that were not identified by the binocular gloss manipulations: this may be due to the very strong image similarity of the images across conditions for the binocular stimuli (Fig. 1*B*). In contrast, dorsal areas V3d, V3A, and V3B/KO were found to respond to both monocular and binocular gloss cues. This pattern suggests that these areas may represent general information about surface gloss regardless of how it is conveyed. Other dorsal areas (especially for hMT+/V5, V7, VIPS, DIPSM, and DIPSA) were engaged by the binocular gloss information but not by monocular gloss cues. Our finding of this dorsal involvement was not anticipated from previous studies of material perception; however, it is broadly consistent with previous imaging studies that have pointed to the strong involvement of dorsal areas in processing binocular cues (Ban et al. 2012; Dövencioğlu et al. 2013; Murphy et al. 2013; Neri et al. 2004; Vanduffel et al. 2002). Higher ventral areas, such as V4 and LO, were also found to be involved in processing binocular gloss information. This is compatible with previous fMRI studies of material perception that have pointed to the involvement of higher ventral areas (Cant and Goodale 2007, 2011; Cavina-Pratesi et al. 2010a, b; Hiramatsu et al. 2011).

It is important to note that slightly different experimental procedures and tasks were used for the binocular and nonstereoscopic gloss sessions. In particular, we used an oddball task for the binocular session to make participants focus on binocular gloss information instead of simply judging on monocular changes (i.e., illumination and object shape), whereas we used a one-back task in the nonstereoscopic session. These differences may have affected the difference of SVM classification performance between the two sessions. However, the performance difference across ROIs within each session should not have been affected. Moreover, the evidence of transfer in V3B/KO, despite differences in procedure, may offer reassurance that this result is likely to be due to the common factors (i.e., gloss) between experiments, rather than differences in task or the 3D shapes.

Although we found clear evidence for fMRI responses that differentiated glossy and nonglossy binocular cues, we did not find activity patterns that supported the decoding of mirror vs. anti-mirror stimuli. From the perspective of the impression of surface material, this is not surprising (these stimuli look equally glossy); however, the stimuli do contain differences in binocular disparities that we might expect the brain to be able to decode. Nevertheless, our stimuli contained disparities that are difficult to fuse (Muryy et al. 2014), perhaps leading to unstable and/or unreliable estimates of binocular disparities. In addition, we presented different shapes that had different disparity structures, meaning that the disparity differences within a shape between mirror and anti-mirrored stimuli may have been overcome by the differences between individual shapes.

We found that the preference for processing information about binocular gloss vs. 3D structure differed across ROIs. In particular, we found that V3B/KO, hMT+/V5, V7, and LO not only responded to binocular gloss information but also information about 3D structure (Fig. 5*A*). The comparison between the Gloss index and the 3D structure index (Fig. 5*B*) shows that V2, DIPSM, DIPSA, and pFs had better classification performance for decoding binocular gloss information than 3D structure information, indicating that these areas may be more specialized for processing surface properties than 3D structure. Interestingly, V2 and pFs were also found to have selectivity for gloss information from specular reflectance in previous studies (Okazawa et al. 2012; Wada et al. 2014), as well as in the current study (Fig. 7). The relatively weaker decoding performance in V2 and pFs for binocularly defined gloss suggests a preference for monocular gloss cues in these areas. By contrast, LO appears to respond to information about binocular gloss and 3D structure equally well (Fig. 5*B*), and most importantly, it was the only ROI that showed a strong transfer effect between the two kinds of information (Fig. 6). One possible explanation is that the processing of binocular gloss and 3D structure influences each other, as shown by previous psychophysical studies (Blake and Bülthoff 1990; Muryy et al. 2013).

A direct means to examine whether an area combines monocular and binocular gloss cues and represents surface gloss in a general way is to test whether the activities that afford classification evoked by one cue type can transfer to the classification of the other. Here, we trained an SVM classifier to discriminate between glossy and matte objects for monocular and binocular gloss information and found transfer effects from monocular to binocular cues in left V3B/KO (as well as a small part of V3v and V1; see Fig. 9). However, we did not find a transfer effect from binocular to monocular gloss cues. A possible explanation for this asymmetry is that the underling neural populations that respond to binocular gloss are more specialized than those that respond to monocular gloss. Under this scenario, we would conceive that a relatively large population of neurons responds to monocular gloss cues, but only a subset of these neurons responds to both monocular and binocular cues. When the classifier is trained on binocular differences, it would select the units that respond to both cues. However, a classifier trained on monocular gloss differences could select voxels reflecting a broad population, many of which do not respond to binocular cues.

More generally, this architecture might suggest that the neural representation of surface material involves a number of colocalized but specialist neuronal populations that respond to a range of different cues that are diagnostic of surface gloss. Previous studies have identified various monocular cues that could contribute to the perception of gloss (Anderson and Kim 2009; Doerschner et al. 2011; Fleming et al. 2003; Gegenfurtner et al. 2013; Kim and Anderson 2010; Kim et al. 2011, 2012; Landy 2007; Marlow and Anderson 2013; Marlow et al. 2011; Motoyoshi et al. 2007; Nishio et al. 2012, 2014; Okazawa et al. 2012; Olkkonen and Brainard 2010; Sun et al. 2015; Wada et al. 2014) and discussed in detail the computations involved in decomposing the intensity gradients in images of surfaces into distinct causes (shading, texture markings, highlights, etc.). Each of these subtypes may be encoded by specialist populations whose aggregated effect supports the impression of gloss. In the case of the binocular gloss cues that we have studied, it seems likely that the brain exploits information about image locations that are difficult to fuse, due to large vertical (ortho-epipolar) disparities or horizontal (epipolar) disparity gradients whose magnitude exceeds fusion limits (Muryy et al. 2013). One means of conceptualizing the differences between the binocular stimuli that we used is in terms of the complexity of the binocular disparity signals; i.e., mirror and anti-mirror stimuli could be thought of as more complex (because of the large disparities) than the painted and flat stimuli. Our results suggest differences between these conditions that align to differences in the perceptual impression of gloss. However, we cannot rule out the possibility that the critical differences related to overall disparity complexity per se rather than gloss. Under this scenario, the areas that we have localized might correspond to a halfway house between a metric based on complexity and one based on the appearance of gloss. Nevertheless, our observation of transfer between monocular and binocular gloss cues is suggestive of a representation of gloss per se.

In summary, we used systematic manipulation of binocular gloss cues to test for cortical areas that respond to surface material properties. We show the involvement of regions within the ventral and dorsal streams and draw direct comparisons with cortical responses defined by monocular gloss cues. Our results point to the potential integration of binocular and monocular cues to material appearance in area V3B/KO that showed partial evidence for transfer between different signals.

## GRANTS

Support for this project was provided by fellowships to A. E. Welchman from the Wellcome Trust (095183/Z/10/Z) and to H. Ban from the Japan Society for the Promotion of Science [JSPS KAKENHI (26870911)].

## DISCLOSURES

No conflicts of interest, financial or otherwise, are declared by the authors.

## AUTHOR CONTRIBUTIONS

H.-C.S., M.D.L., and A.E.W. conception and design of research; H.-C.S. performed experiments; H.-C.S. and H.B. analyzed data; H.-C.S., M.D.L., H.B., and A.E.W. interpreted results of experiments; H.-C.S., A.M., and A.E.W. prepared figures; H.-C.S. drafted manuscript; H.-C.S., M.D.L., H.B., R.W.F., and A.E.W. edited and revised manuscript; H.-C.S., M.D.L., H.B., A.M., R.W.F., and A.E.W. approved final version of manuscript.

## References

[B1] Anderson BL Visual perception of materials and surfaces. Curr Biol 21: R978–R983, 2011. 22192826 10.1016/j.cub.2011.11.022

[B2] Anderson BL, Kim J Image statistics do not explain the perception of gloss and lightness. J Vis 10: 3, 2009. 10.1167/9.11.10 20053073

[B3] Ban H, Preston TJ, Meeson A, Welchman AE The integration of motion and disparity cues to depth in dorsal visual cortex. Nat Neurosci 15: 636–643, 2012. 22327475 10.1038/nn.3046 PMC3378632

[B4] Ban H, Yamamoto H A non-device-specific approach to display characterization based on linear, nonlinear, and hybrid search algorithms. J Vis 13: 20, 2013. 23729771 10.1167/13.6.20

[B5] Blake A, Bülthoff H Does the brain know the physics of specular reflection? Nature 343: 165–168, 1990. 2296307 10.1038/343165a0

[B6] Brainard DH The Psychophysics Toolbox. Spat Vis 10: 433–436, 1997. 9176952

[B7] Cant JS, Goodale MA Attention to form or surface properties modulates different regions of human occipitotemporal cortex. Cereb Cortex 17: 713–731, 2007. 16648452 10.1093/cercor/bhk022

[B8] Cant JS, Goodale MA Scratching beneath the surface: new insights into the functional properties of the lateral occipital area and parahippocampal place area. J Neurosci 31: 8248–8258, 2011. 21632946 10.1523/JNEUROSCI.6113-10.2011 PMC6622867

[B9] Cavina-Pratesi C, Kentridge RW, Heywood CA, Milner AD Separate channels for processing form, texture, and color: evidence from fMRI adaptation and visual object agnosia. Cereb Cortex 20: 2319–2332, 2010a. 20100900 10.1093/cercor/bhp298

[B10] Cavina-Pratesi C, Kentridge RW, Heywood CA, Milner AD Separate processing of texture and form in the ventral stream: evidence from fMRI and visual agnosia. Cereb Cortex 20: 433–446, 2010b. 19478035 10.1093/cercor/bhp111

[B11] Chang CC, Lin CJ LIBSVM: a library for support vector machines. ACM Trans Intell Syst Technol 2: 27, 2011.

[B12] Debevec P Rendering synthetic objects into real scenes: bridging traditional and image-based graphics with global illumination and high dynamic range photography. In: Proceedings of the 25th Annual Conference on Computer Graphics and Interactive Techniques. Orlando, FL: 1998.

[B13] Doerschner K, Fleming RW, Yilmaz O, Schrater PR, Hartung B, Kersten D Visual motion and the perception of surface material. Curr Biol 21: 2010–2016, 2011. 22119529 10.1016/j.cub.2011.10.036 PMC3246380

[B14] Doerschner K, Maloney LT, Boyaci H Perceived glossiness in high dynamic range scenes. J Vis 10: 11, 2010. 20936748 10.1167/10.9.11 PMC4455063

[B15] Dövencioğlu D, Ban H, Schofield AJ, Welchman AE Perceptual integration for qualitatively different 3-D cues in the human brain. J Cogn Neurosci 25: 1527–1541, 2013. 23647559 10.1162/jocn_a_00417 PMC3785137

[B16] Fleming RW, Dror RO, Adelson EH Real-world illumination and the perception of surface reflectance properties. J Vis 3: 346–368, 2003. 10.1167/3.5.3 12875632

[B17] Gegenfurtner K, Baumgartner E, Wiebel C The perception of gloss in natural images. J Vis 13: 200, 2013.

[B18] Hiramatsu C, Goda N, Komatsu H Transformation from image-based to perceptual representation of materials along the human ventral visual pathway. Neuroimage 57: 482–494, 2011. 21569854 10.1016/j.neuroimage.2011.04.056

[B19] Junghöfer M, Schupp HT, Stark R, Vaitl D Neuroimaging of emotion: empirical effects of proportional global signal scaling in fMRI data analysis. Neuroimage 25: 520–526, 2005. 15784431 10.1016/j.neuroimage.2004.12.011

[B20] Kentridge RW, Thomson R, Heywood CA Glossiness perception can be mediated independently of cortical processing of colour or texture. Cortex 48: 1244–1246, 2012. 22402337 10.1016/j.cortex.2012.01.011

[B21] Kerrigan IS, Adams WJ Highlights, disparity, and perceived gloss with convex and concave surfaces. J Vis 13: 9, 2013. 23291649 10.1167/13.1.9

[B22] Kim J, Anderson BL Image statistics and the perception of surface gloss and lightness. J Vis 10: 3, 2010. 10.1167/10.9.3 20884601

[B23] Kim J, Marlow P, Anderson BL The perception of gloss depends on highlight congruence with surface shading. J Vis 11: pii: 4, 2011. 21841140 10.1167/11.9.4

[B24] Kim J, Marlow PJ, Anderson BL The dark side of gloss. Nat Neurosci 15: 1590–1595, 2012. 23001059 10.1038/nn.3221

[B25] Kriegeskorte N, Goebel R, Bandettini P Information-based functional brain mapping. Proc Natl Acad Sci USA 103: 3863–3868, 2006. 16537458 10.1073/pnas.0600244103 PMC1383651

[B26] Landy MS Visual perception: a gloss on surface properties. Nature 447: 158–159, 2007. 17443194 10.1038/nature05714 PMC2745613

[B27] Marlow P, Anderson BL Generative constraints on image cues for perceived gloss. J Vis 13: pii: 2, 2013. 24297776 10.1167/13.14.2

[B28] Marlow P, Kim J, Anderson BL The perception and misperception of specular surface reflectance. Curr Biol 22: 1909–1913, 2012. 22959347 10.1016/j.cub.2012.08.009

[B29] Marlow P, Kim J, Anderson BL The role of brightness and orientation congruence in the perception of surface gloss. J Vis 11: pii: 16, 2011. 21873616 10.1167/11.9.16

[B30] Motoyoshi I, Nishida S, Sharan L, Adelson EH Image statistics and the perception of surface qualities. Nature 447: 206–209, 2007. 17443193 10.1038/nature05724

[B31] Murphy AP, Ban H, Welchman AE Integration of texture and disparity cues to surface slant in dorsal visual cortex. J Neurophysiol 110: 190–203, 2013. 23576705 10.1152/jn.01055.2012 PMC3727040

[B32] Muryy AA, Fleming RW, Welchman AE Binocular cues for glossiness. J Vis 12: 869, 2012.

[B33] Muryy AA, Fleming RW, Welchman AE Key characteristics of specular stereo. J Vis 14: 1–26, 2014. 10.1167/14.14.14 PMC4278431 25540263

[B34] Muryy AA, Welchman AE, Blake A, Fleming RW Specular reflections and the estimation of shape from binocular disparity. Proc Natl Acad Sci USA 110: 2413–2418, 2013. 23341602 10.1073/pnas.1212417110 PMC3568321

[B35] Neri P, Bridge H, Heeger DJ Stereoscopic processing of absolute and relative disparity in human visual cortex. J Neurophysiol 92: 1880–1891, 2004. 15331652 10.1152/jn.01042.2003

[B36] Nishio A, Goda N, Komatsu H Neural selectivity and representation of gloss in the monkey inferior temporal cortex. J Neurosci 32: 10780–10793, 2012. 22855825 10.1523/JNEUROSCI.1095-12.2012 PMC6621409

[B37] Nishio A, Shimokawa T, Goda N, Komatsu H Perceptual gloss parameters are encoded by population responses in the monkey inferior temporal cortex. J Neurosci 34: 11143–11151, 2014. 25122910 10.1523/JNEUROSCI.1451-14.2014 PMC6705264

[B38] Obein G, Knoblauch K, Viénot F Difference scaling of gloss: nonlinearity, binocularity, and constancy. J Vis 4: 711–720, 2004. 15493965 10.1167/4.9.4

[B39] Okazawa G, Goda N, Komatsu H Selective responses to specular surfaces in the macaque visual cortex revealed by fMRI. Neuroimage 63: 1321–1333, 2012. 22885246 10.1016/j.neuroimage.2012.07.052

[B40] Olkkonen M, Brainard DH Perceived glossiness and lightness under real-world illumination. J Vis 10: 5, 2010. 20884603 10.1167/10.9.5 PMC2981171

[B41] Orban GA, Claeys K, Nelissen K, Smans R, Sunaert S, Todd JT, Wardak C, Durand JB, Vanduffel W Mapping the parietal cortex of human and non-human primates. Neuropsychologia 44: 2647–2667, 2006. 16343560 10.1016/j.neuropsychologia.2005.11.001

[B42] Orban GA, Fize D, Peuskens H, Denys K, Nelissen K, Sunaert S, Todd J, Vanduffel W Similarities and differences in motion processing between the human and macaque brain: evidence from fMRI. Neuropsychologia 41: 1757–1768, 2003. 14527539 10.1016/s0028-3932(03)00177-5

[B43] Orban GA, Sunaert S, Todd JT, Van Hecke P, Marchal G Human cortical regions involved in extracting depth from motion. Neuron 24: 929–940, 1999. 10624956 10.1016/s0896-6273(00)81040-5

[B44] Pelli DG The VideoToolbox software for visual psychophysics: transforming numbers into movies. Spat Vis 10: 437–442, 1997. 9176953

[B45] Preston TJ, Kourtzi Z, Welchman AE Adaptive estimation of three-dimensional structure in the human brain. J Neurosci 29: 1688–1698, 2009. 19211876 10.1523/JNEUROSCI.5021-08.2009 PMC6666271

[B46] Sakano Y, Ando H Effects of head motion and stereo viewing on perceived glossiness. J Vis 10: 15, 2010. 21106677 10.1167/10.9.15

[B47] Sharan L, Li Y, Motoyoshi I, Nishida S, Adelson EH Image statistics for surface reflectance perception. J Opt Soc Am A Opt Image Sci Vis 25: 846–865, 2008. 18382484 10.1364/josaa.25.000846

[B48] Sun HC, Ban H, Di Luca M, Welchman AE fMRI evidence for areas that process surface gloss in the human visual cortex. Vision Res 109: 149–157, 2015. 25490434 10.1016/j.visres.2014.11.012 PMC4410797

[B49] Sun HC, Welchman AE, Chang DH, Di Luca M Look but don't touch: visual cues to surface structure drive somatosensory cortex. Neuroimage 128: 353–361, 2016. 26778128 10.1016/j.neuroimage.2015.12.054 PMC4767223

[B50] Vanduffel W, Fize D, Peuskens H, Denys K, Sunaert S, Todd JT, Orban GA Extracting 3D from motion: differences in human and monkey intraparietal cortex. Science 298: 413–415, 2002. 12376701 10.1126/science.1073574

[B51] Wada A, Sakano Y, Ando H Human cortical areas involved in perception of surface glossiness. Neuroimage 98: 243–257, 2014. 24825505 10.1016/j.neuroimage.2014.05.001

[B52] Wendt G, Faul F, Ekroll V, Mausfeld R Disparity, motion, and color information improve gloss constancy performance. J Vis 10: 7, 2010. 20884605 10.1167/10.9.7

[B53] Wendt G, Faul F, Mausfeld R Highlight disparity contributes to the authenticity and strength of perceived glossiness. J Vis 8: 14, 2008. 18318617 10.1167/8.1.14

